# Prevalence of antibiotic resistance in *Salmonella* Typhimurium isolates originating from Iran: a systematic review and meta-analysis

**DOI:** 10.3389/fvets.2024.1388790

**Published:** 2024-05-27

**Authors:** Negar Narimisa, Shabnam Razavi, Faramarz Masjedian Jazi

**Affiliations:** Department of Microbiology, School of Medicine, Iran University of Medical Sciences, Tehran, Iran

**Keywords:** *Salmonella* Typhimurium, antibiotic resistance, tetracycline, cefixime, meta-analysis

## Abstract

**Objective:**

Antibiotic resistance in *Salmonella* represents a significant global public health concern. Among various serovars, *Salmonella* enterica serovar Typhimurium is prevalent in multiple countries. This study aims to conduct a systematic review and meta-analysis to evaluate the pattern of antibiotic resistance in *S*. Typhimurium isolates from diverse sources in Iran.

**Methods:**

We conducted a comprehensive and systematic search for relevant articles until December 2023 in the following databases: PubMed, Scopus, Web of Science, and SID. The collected data were analyzed using Stata software version 17.

**Results:**

Eighteen studies examined the pattern of antibiotic resistance in *S*. Typhimurium for various antibiotics in Iran. Piperacillin and tetracycline exhibited the highest resistance rates, at 79 and 60% respectively, while cefixime and ceftriaxone had the lowest resistance rates at 0%.

**Conclusion:**

Our findings indicate a high level of antibiotic resistance among the studied antibiotics. This high level of antibiotic resistance raises concerns and underscores the necessity for monitoring the use of antibiotics. Moreover, resistance to these antibiotics was more prevalent in samples isolated from animals compared to other sources. This highlights the importance of animal screening to detect the presence of drug-resistant isolates, with the ultimate goal of reducing antibiotic resistance and preventing the transmission of resistant strains to humans.

## Introduction

Salmonellosis is one of the most common food-borne diseases worldwide, causing severe and occasionally deadly infections in humans (1). Based on published data, over 2,500 serotypes of *Salmonella* have been recognized, with 1,500 serovars linked to human and animal diseases (2).

*Salmonella* serovars have the ability to infect a diverse array of domestic animals such as sheep, cattle, poultry, and pigs. Infected animals may exhibit varying symptoms, varying from mild gastroenteritis to severe infections that can be fatal (3). Painter et al. ~17.9% of foodborne illnesses are linked to poultry, with 19% of these poultry-related illnesses attributed to *Salmonella enterica* (3).

Non-typhoidal salmonellosis is an important enteric infection in humans, particularly in neonates and young children (4). The consumption of contaminated animal-derived foods such as beef, poultry, pork, and lamb is the major route of *Salmonella* transmission to humans (5).

Non-typhoidal *Salmonella* causes ~153 million cases of gastroenteritis and 57,000 deaths annually worldwide (6). *Salmonella* Typhimurium has been reported as one of the most widespread foodborne pathogens in many countries (7). This serovar is the most common in Europe and can be isolated from humans, pigs, and pork. In the United States, it is one of five serovars associated with human salmonellosis (7, 8).

One of the recent global public health concerns regarding salmonellosis is the emergence and spread of resistant *Salmonella* strains, including multiple drug-resistant (MDR) strains in both humans and animals (9).

Antibiotics are not commonly used to treat human salmonellosis, as the illness typically resolves in 5–7 days without treatment (10). However, in some cases, antibiotic therapy may be necessary (10). In these instances, appropriate antimicrobial therapy such as ciprofloxacin in adults and ceftriaxone in children can be used (4, 10). Nonetheless, treating these patients can sometimes be challenging due to antibiotic resistance of pathogen (11).

The use of antimicrobial agents in any environment creates pressures that favor the survival of antibiotic-resistant pathogens (12). The routine practice of administering antimicrobial agents to domestic livestock for disease prevention, treatment, and growth promotion is a significant factor in the emergence of antibiotic-resistant bacteria, which are subsequently transmitted to humans through the food chain (13, 14). Most infections with antimicrobial-resistant *Salmonella* are acquired by eating contaminated foods of animal origin (15).

Therefore, this meta-analysis was conducted to investigate the prevalence of antimicrobial resistance of *S*. Typhimurium isolates in Iran. Additionally, this study provides a better understanding of antibiotic resistance in this bacterium, ultimately helping to choose the most optimal and effective treatment approach.

## Materials and methods

### Search strategy

We conducted a comprehensive search in the PubMed, Web of Science, Scopus, and Scientific Information Database (SID) databases up to December 2023 to identify potentially relevant studies. The search criteria used were (“*Salmonella* Typhimurium” OR “*S*. Typhimurium”) AND (Resistan^*^ OR suscep^*^) AND (Iran) with their respective Mesh terms.

### Inclusion and exclusion criteria

All original articles that reported the prevalence of antibiotic resistance among *S*. Typhimurium isolates in Iran were included. Articles were excluded if they were reviews, conference presentations, case reports, studies with unclear results, or written in languages other than English and Persian.

### Data extraction

After consolidating the articles in the EndNote X20 Citation Manager Software, duplicate articles were removed before the review process. The citations were then uploaded to Rayyan, a citation classification application (16). Two independent reviewers screened the titles and abstracts and removed irrelevant articles. The full texts of potentially relevant articles were collected and reviewed independently by two authors. Data from eligible studies, including the first author, publication year, source of samples, sample size (total isolates and number of resistances of *S*. Typhimurium for various antibiotics), were independently extracted by two researchers. Inconsistencies between the reviewers were resolved through discussion to reach a consensus.

### Quality assessment of studies

The quality of the included studies was assessed using the Joanna Briggs Institute (JBI) checklist (17). Two reviewers independently evaluated and scored the quality of each article included in the review, resolving any disagreements through discussion.

### Statistical analysis

The meta-analysis and generation of forest plots for this review were conducted using Stata 17 software. The *I*-squared index (*I*^2^) was used to assess the possibility of heterogeneity among studies, categorizing the degree of heterogeneity as low, moderate, or high based on *I*^2^-values (expressed as percentages around 25, 50, and 75, respectively). To evaluate publication bias, Egger and Begg tests were performed; a significance level of *P* < 0.05 indicated statistically significant publication bias. The pooled prevalence of *S*. Typhimurium resistance to the investigated antibiotics, along with corresponding 95% confidence intervals (CIs), was calculated using forest plots in a random-effects model. Moreover, subgroup meta-analysis was conducted for the source of *S*. Typhimurium isolates for different antibiotics if a sufficient number of articles were available.

## Results

### Characteristics of the included studies

**Figure 1:**
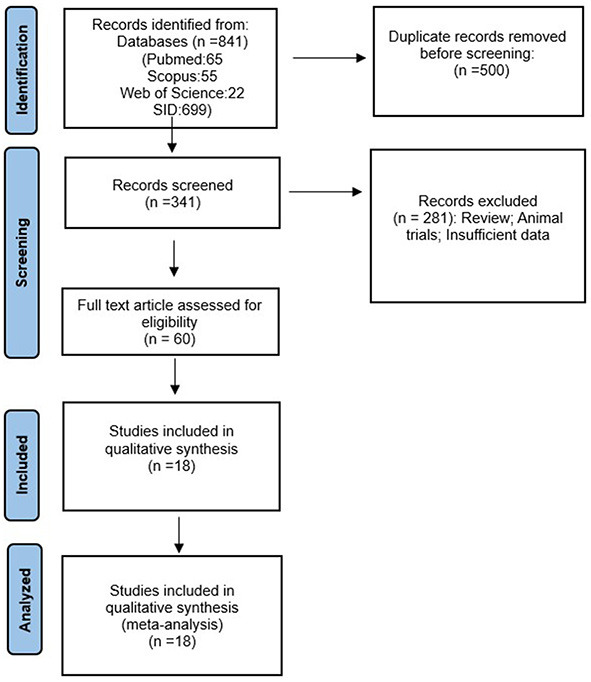
The study Prisma flow diagram.

A total of 841 articles were obtained through a database search. After eliminating duplicates, 500 articles remained. Subsequently, 60 publications underwent full-text evaluation. As a result, 42 articles were excluded, and this study included 18 cross-sectional studies that examined the antibiotic resistance of *S*. Typhimurium (Figure 1). The characteristics of the included studies are presented in Table 1. Additionally, Table 2 contains the results of the quality assessment.

**Table 1:** Characteristics of included studies.

**References**	**City**	**Source**	**Total**	**Method**
Askari et al. (18)	Tehran	Animal	7	E-test
Azizpour et al. (19)	Ardebil	Animal	1	Disk diffusion
Doosti et al. (20)	Chaharmahal va Bakhtiari	Animal	138	Disk diffusion
Ezatpanah et al. (21)	Arak	Animal	4	Disk diffusion
Firoozeh et al. (22)	Tehran	Human	5	Disk diffusion
Hosseinzadeh et al. (23)	Tehran	Animal	23	Disk diffusion
Amani et al. (24)	Tehran		35	Disk diffusion
Keshmiri et al. (25)	Various		11	Disk diffusion
Akbari Khakrizi et al. (26)	Tehran	Animal	4	Disk diffusion
Manafi et al. (27)	Urmia	Food	3	Disk diffusion
Yousefi-Mashouf and Moshtaghi (28)	Hamadan	Human	22	Disk diffusion
Mehrabian and Jaberi (29)	Tehran	Food	12	Disk diffusion
Nazari Moghadam et al. (30)	Chaharmahal va Bakhtiari	Food	36	Disk diffusion
Farhoudi-Moghadam et al. (31)	Tehran	Human	232	Agar dilution
Monadi et al. (32)	Kohgiluyeh and Boyer Ahmad	Food	12	Disk diffusion
Nazer and Safari (33)	Shiraz	Food	10	Disk diffusion
Rahimi et al. (34)	Various	Animal	4	Disk diffusion
Ranjbar et al. (35)	Tehran	Human	21	Disk diffusion

**Table 2:** Quality score of included studies.

**References**	**Q1**	**Q2**	**Q3**	**Q4**	**Q5**	**Q6**	**Q7**	**Q8**	**Q9**	**Total score**
Askari et al. (18)	Y	Y	Y	Y	Y	Y	N	N	Y	7
Azizpour et al. (19)	Y	Y	Y	Y	Y	Y	Y	Y	Y	9
Doosti et al. (20)	Y	Y	Y	Y	Y	Y	Y	Y	Y	9
Ezatpanah et al. (21)	Y	Y	Y	Y	Y	Y	Y	Y	N	8
Firoozeh et al. (22)	Y	Y	N	Y	Y	Y	Y	Y	N	7
Hosseinzadeh et al. (23)	U	Y	U	Y	N	Y	Y	Y	N	5
Amani et al. (24)	Y	Y	Y	Y	Y	Y	Y	Y	Y	9
Keshmiri et al. (25)	N	Y	Y	N	Y	Y	Y	Y	Y	7
Akbari Khakrizi et al. (26)	Y	Y	Y	Y	Y	Y	Y	Y	Y	9
Manafi et al. (27)	Y	Y	Y	Y	Y	Y	Y	Y	Y	9
Yousefi-Mashouf and Moshtaghi (28)	Y	Y	Y	Y	N	Y	Y	Y	Y	8
Mehrabian and Jaberi (29)	N	Y	Y	Y	Y	Y	N	N	Y	6
Nazari Moghadam et al. (30)	Y	Y	Y	Y	Y	Y	N	Y	Y	8
Farhoudi-Moghadam et al. (31)	Y	Y	N	Y	Y	Y	Y	Y	N	7
Monadi et al. (32)	Y	Y	Y	Y	Y	Y	Y	Y	Y	9
Nazer and Safari (33)	Y	Y	N	Y	Y	Y	N	Y	N	6
Rahimi et al. (34)	Y	Y	Y	Y	Y	Y	Y	Y	Y	9
Ranjbar et al. (35)	N	Y	N	Y	Y	Y	Y	Y	N	6

Total score is from 1 (lowest) to 9 (highest); Q = question; Y = Yes; N = No; U = Unclear. Q1: Was the sample frame appropriate to address the target population? Q2: Were study participants sampled in an appropriate way? Q3: Was the sample size adequate? Q4: Were the study subjects and the setting described in detail? Q5: Was the data analysis conducted with sufficient coverage of the identified sample? Q6: Were valid methods used for the identification of the condition? Q7: Was the condition measured in a standard, reliable way for all participants? Q8: Was there appropriate statistical analysis? Q9: Was the response rate adequate, and if not, was the low response rate managed appropriately?

### Data analysis

#### Prevalence of cephalosporin resistance

**Figure 2:**
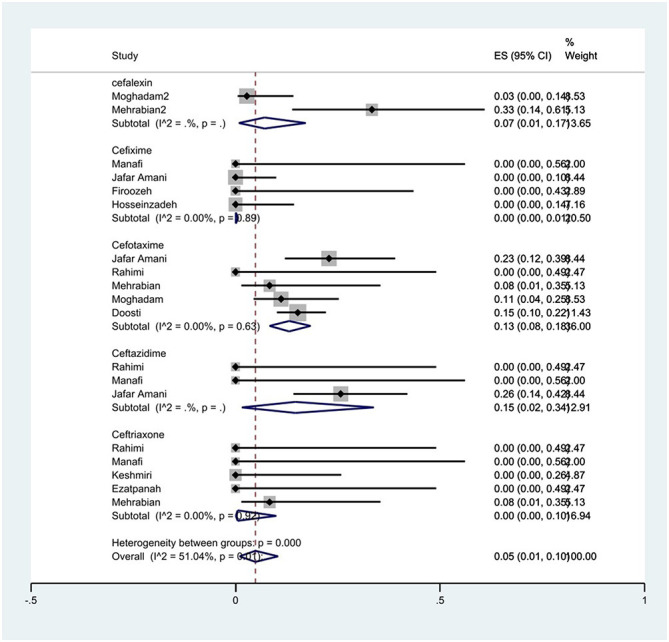
Forest plot showing the prevalence of cephalosporin family resistance of *S*. Typhimurium in Iran.

The susceptibility to cefalexin was determined in two studies. The prevalence of cefalexin resistance was 7% (95% CI: 1–17%). The susceptibility to cefixime was determined in four studies. The prevalence of cefixime resistance was 0% (95% CI: 0–1%) with substantial heterogeneity (*I*^2^ = 0%, *P* = 0.89). The susceptibility to cefotaxime was determined in five studies. The prevalence of cefotaxime resistance was 13% (95% CI: 8–18%) with substantial heterogeneity (*I*^2^ = 0%, *P* = 0.63). The susceptibility to ceftazidime was determined in three studies. The prevalence of ceftazidime resistance was 15% (95% CI: 2–34%). The susceptibility to ceftriaxone was determined in five studies. The prevalence of ceftriaxone resistance was 0% (95% CI: 0–10%) with substantial heterogeneity (*I*^2^ = 0%, *P* = 0.93; Figure 2).

#### Prevalence of penicillin resistance

**Figure 3:**
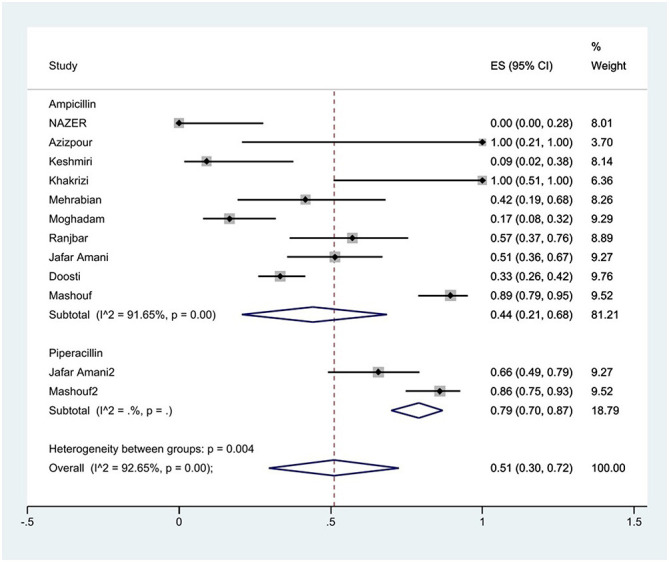
Forest plot showing the prevalence of penicillin family resistance of *S*. Typhimurium in Iran.

**Figure 4:**
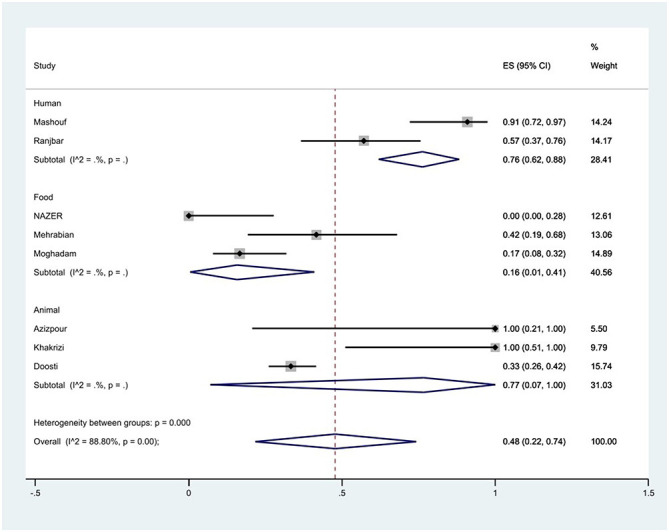
Forest plot showing the prevalence of ampicillin resistance of *S*. Typhimurium in Iran from different sources.

The susceptibility to piperacillin was determined in two studies. The prevalence of piperacillin resistance was 79% (95% CI: 70–87%). The susceptibility to ampicillin was determined in 10 studies. The prevalence of ampicillin resistance was 44% (95% CI: 21–68%) with substantial heterogeneity (*I*^2^ = 91.65%, *P* < 0.01; Figure 3). Furthermore, subgroup meta-analysis of ampicillin resistance based on the source of *S*. Typhimurium isolation showed that the rate of ampicillin resistance in animal samples was 77% (95% CI: 7–100%), which was higher than in food and human sources (*P* < 0.01; Figure 4).

#### Prevalence of fluoroquinolones resistance

**Figure 5:**
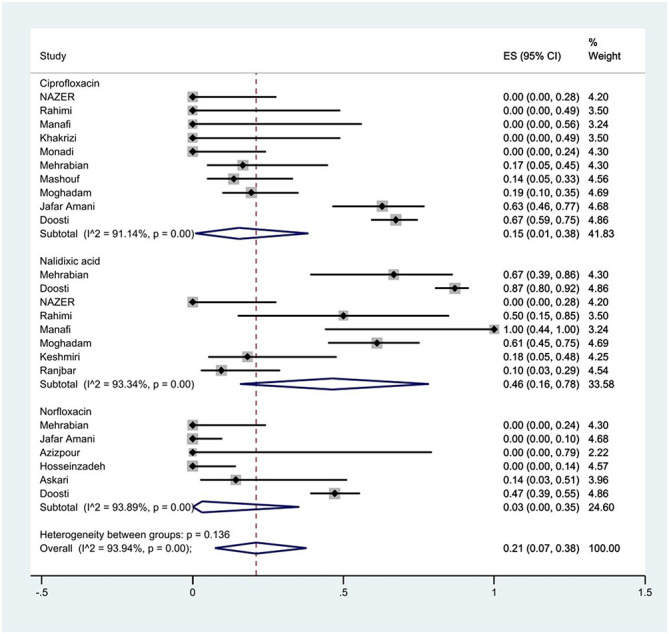
Forest plot showing the prevalence of fluoroquinolones family resistance of *S*. Typhimurium in Iran.

**Figure 6:**
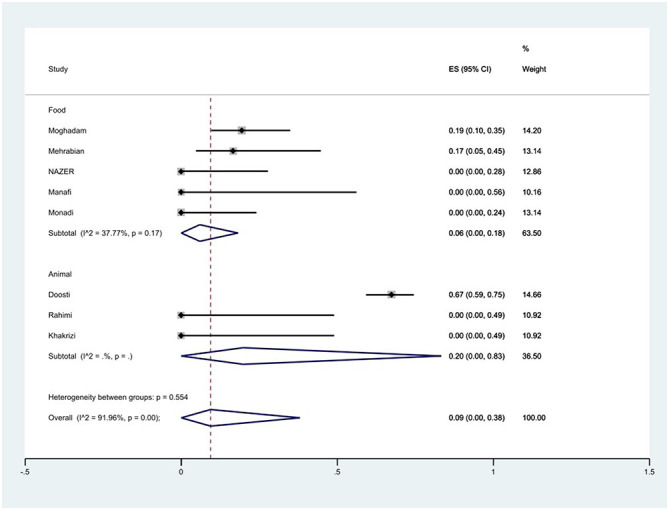
Forest plot showing the prevalence of ciprofloxacin resistance of *S*. Typhimurium in Iran from different sources.

**Figure 7:**
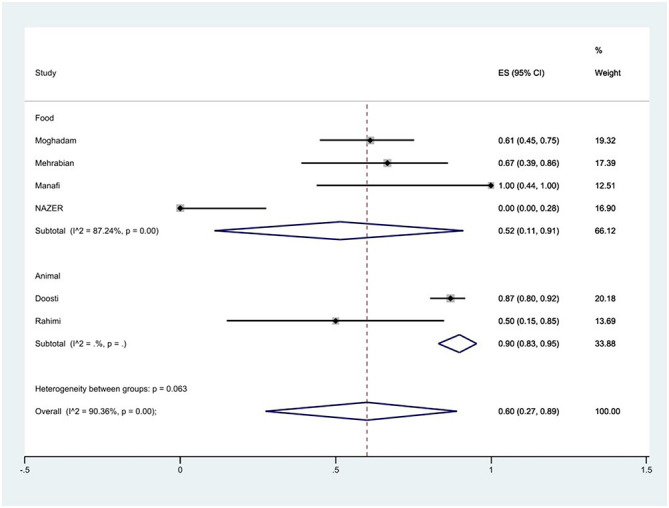
Forest plot showing the prevalence of nalidixic acid resistance of *S*. Typhimurium in Iran from different sources.

The susceptibility to norfloxacin was determined in six studies. The prevalence of norfloxacin resistance was 3% (95% CI: 0–35%) with substantial heterogeneity (*I*^2^ = 93.89%, *P* < 0.01; Figure 5). The susceptibility to ciprofloxacin was determined in ten studies. The prevalence of ciprofloxacin resistance was 15% (95% CI: 1–38%) with substantial heterogeneity (*I*^2^ = 91.14%, *P* < 0.01). Additionally, subgroup meta-analysis of ciprofloxacin resistance based on the source of *S*. Typhimurium isolation showed that the rate of ciprofloxacin resistance in animal samples was 20% (95% CI: 0–83%), which was higher than in food sources (*P* = 0.554; Figure 6). The susceptibility to nalidixic acid was determined in eight studies. The prevalence of nalidixic acid resistance was 46% (95% CI: 16–78%) with substantial heterogeneity (*I*^2^ = 93.89%, *P* < 0.01). In addition, subgroup meta-analysis of nalidixic acid resistance based on the source of *S*. Typhimurium isolation showed that the rate of nalidixic acid resistance in animal samples was 90% (95% CI: 83–96%), which was higher than in food sources (*P* = 0.063; Figure 7).

#### Prevalence of tetracycline resistance

**Figure 8:**
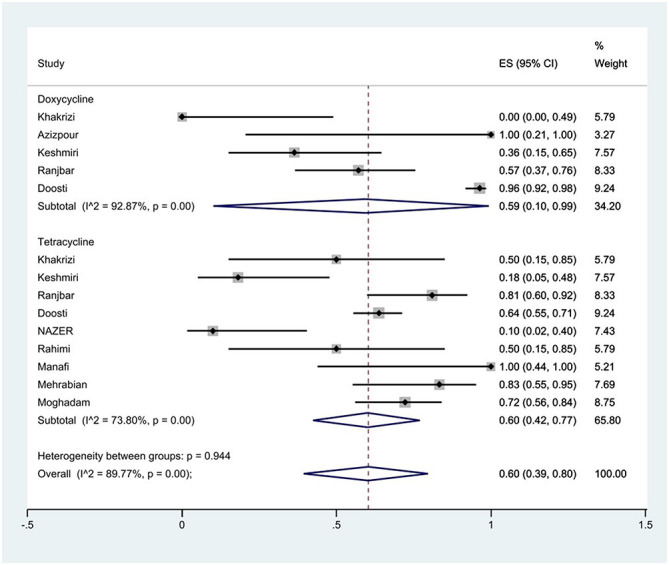
Forest plot showing the prevalence of tetracycline family resistance of *S*. Typhimurium in Iran.

**Figure 9:**
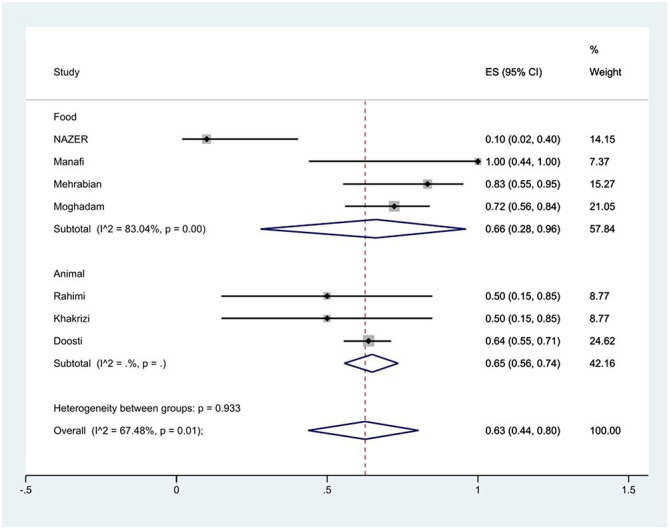
Forest plot showing the prevalence of tetracycline resistance of *S*. Typhimurium in Iran from different sources.

The susceptibility to doxycycline was determined in five studies. The prevalence of doxycycline resistance was 59% (95% CI: 10–99%) with substantial heterogeneity (*I*^2^ = 92.87%, *P* < 0.01; Figure 8). The susceptibility to tetracycline was determined in nine studies. The prevalence of tetracycline resistance was 60% (95% CI: 42–77%) with substantial heterogeneity (*I*^2^ = 73.80%, *P* < 0.01). Moreover, subgroup meta-analysis of tetracycline resistance based on the source of *S*. Typhimurium isolation showed that the rate of tetracycline resistance was similar in animal samples at 65% (95% CI: 56–74%) and food samples at 66% (95% CI: 28–96%; *P* = 0.933; Figure 9).

#### Prevalence of aminoglycoside resistance

**Figure 10:**
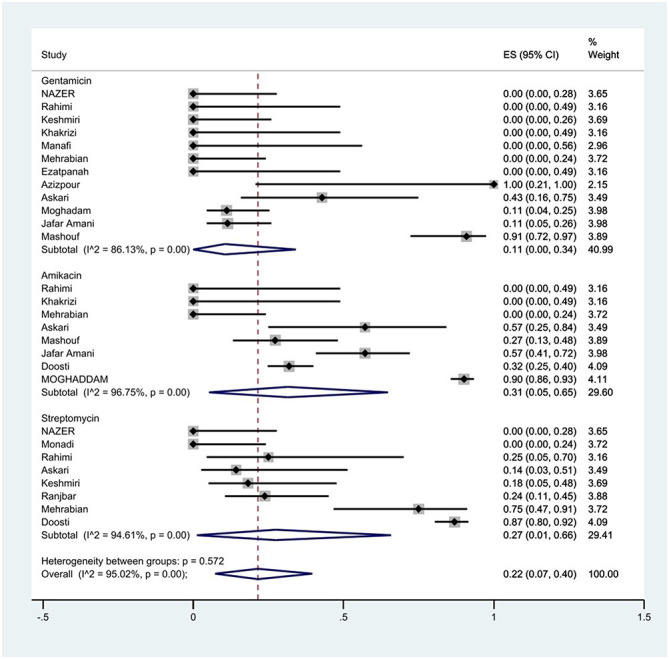
Forest plot showing the prevalence of aminoglycoside family resistance of *S*. Typhimurium in Iran.

The susceptibility to amikacin was determined in eight studies. The prevalence of amikacin resistance was 31% (95% CI: 5–65%) with substantial heterogeneity (*I*^2^ = 96.75%, *P* < 0.01; Figure 10).

The susceptibility to gentamicin was determined in 12 studies. The prevalence of gentamicin resistance was 11% (95% CI: 0–34%) with substantial heterogeneity (*I*^2^ = 86.13%, *P* < 0.01).

**Figure 11:**
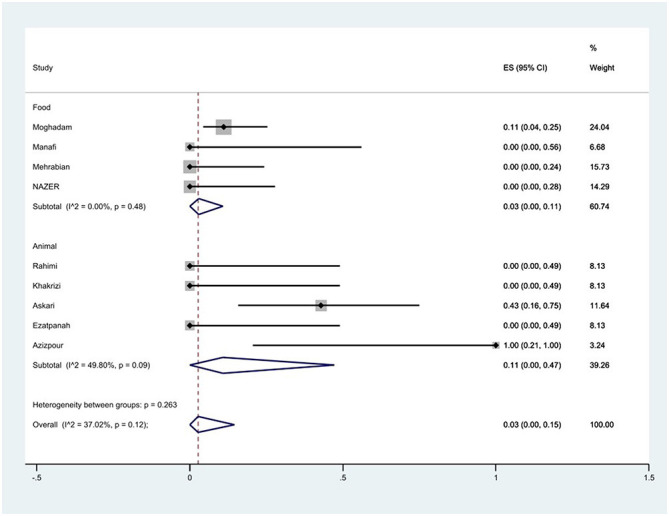
Forest plot showing the prevalence of gentamicin resistance of *S*. Typhimurium in Iran from different sources.

Furthermore, a subgroup meta-analysis of gentamicin resistance based on the source of *S*. Typhimurium isolation indicated that the rate of gentamicin resistance in animal samples was 11% (95% CI: 0–47%), which was higher than that in food sources (*P* = 0.263; Figure 11).

The susceptibility to streptomycin was determined in eight studies. The prevalence of streptomycin resistance was 27% (95% CI: 1–66%) with substantial heterogeneity (*I*^2^ = 94.61%, *P* < 0.01).

**Figure 12:**
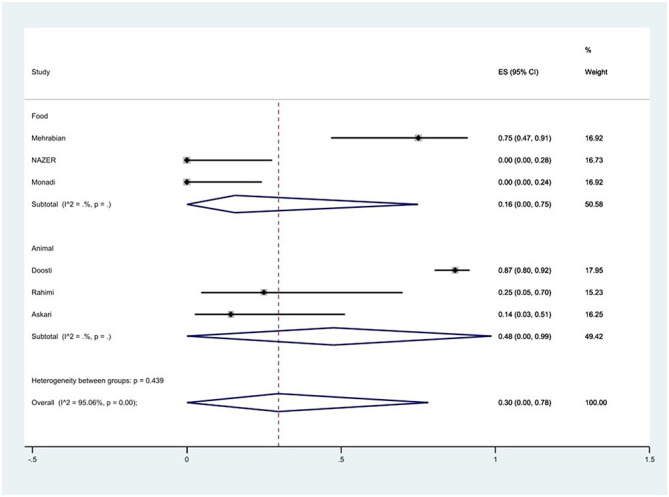
Forest plot showing the prevalence of streptomycin resistance of *S*. Typhimurium in Iran from different sources.

Additionally, a subgroup meta-analysis of streptomycin resistance based on the source of *S*. Typhimurium isolation demonstrated that the rate of streptomycin resistance in animal samples was 48% (95% CI: 0–99%), which was higher than in food sources (*P* = 0.439; Figure 12).

#### Prevalence of chloramphenicol resistance

**Figure 13:**
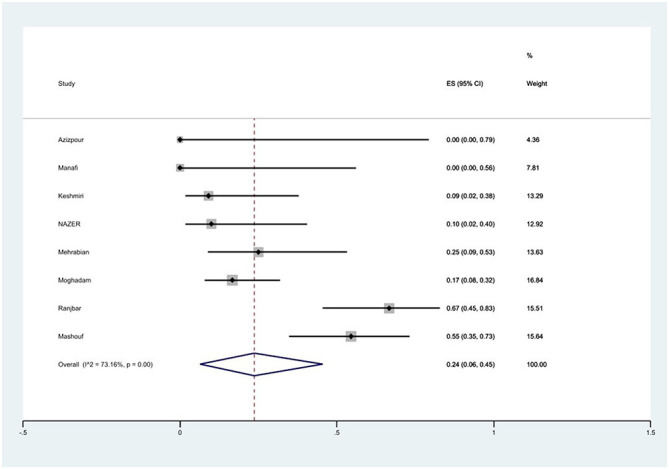
Forest plot showing the prevalence of chloramphenicol resistance of *S*. Typhimurium in Iran.

The susceptibility to chloramphenicol was determined in eight studies. The prevalence of chloramphenicol resistance was 24% (95% CI: 6–45%) with substantial heterogeneity (*I*^2^ = 73.16%, *P* < 0.01; Figure 13).

#### Prevalence of imipenem resistance

**Figure 14:**
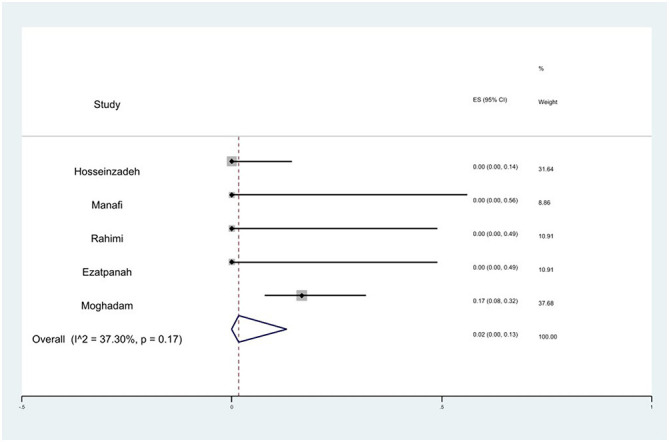
Forest plot showing the prevalence of imipenem resistance of *S*. Typhimurium in Iran.

The susceptibility to imipenem was determined in five studies. The prevalence of imipenem resistance was 2% (95% CI: 0–13%) with substantial heterogeneity (*I*^2^ = 37.30%, *P* = 0.17; Figure 14).

#### Prevalence of trimethoprim/sulfamethoxazole (SXT) resistance

**Figure 15:**
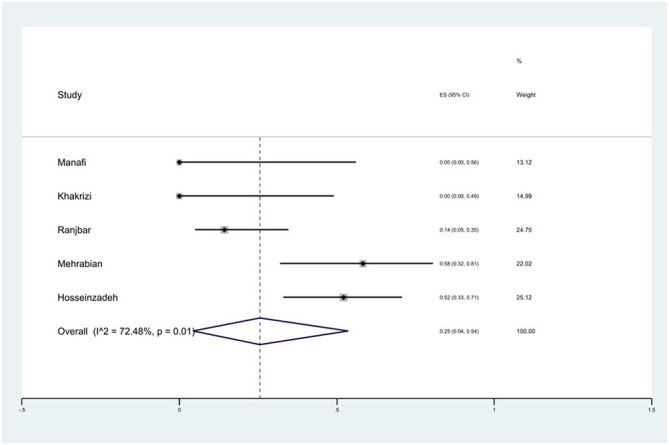
Forest plot showing the prevalence of SXT resistance of *S*. Typhimurium in Iran.

The susceptibility to SXT was determined in five studies. The prevalence of SXT resistance was 25% (95% CI: 4–54%) with substantial heterogeneity (*I*^2^ = 72.48%, *P* = 0.01; Figure 15).

## Discussion

Recently, there have been reports of an increase in the prevalence of *Salmonella* strains that are resistant to antimicrobial agents (36). This surge in prevalence poses a significant public health concern (27). Therefore, acquiring epidemiological information on drug resistance can assist physicians and healthcare professionals in selecting appropriate antimicrobials and averting treatment failure. Given that serovar Typhimurium is among the prevalent *Salmonella* serovars in numerous countries, we utilized a meta-analysis approach to gather pertinent published data on the antibiotic resistance patterns of *S*. Typhimurium isolates in Iran.

Our results showed that piperacillin, tetracycline, doxycycline, nalidixic acid, and ampicillin had the highest resistance rates in *S*. Typhimurium isolates in Iran, with 79, 60, 59, 46, and 44%, respectively. The high rate of antibiotic resistance for these antibiotics may be the result of improper use or misuse of these antibiotics in human clinical treatment and animal food production (37). Improper use of antibiotics may lead to exposure of bacteria to antibiotics, which increases the possibility of antibiotic-resistant bacteria (12). In general, the increase in the antibiotic resistance levels of *S*. Typhimurium isolates may contribute to the further spread of the serovar (38), which highlights the importance of establishing targeted surveillance throughout the country to obtain more information about the antibiotic resistance of this bacterium in order to take appropriate measures to reduce its prevalence in different sources.

While cefixime, ceftriaxone, imipenem, and norfloxacin antibiotics were the most effective against *S*. Typhimurium with 0, 0, 2, and 3% resistance, respectively. Therefore, these drugs could be applied as treatment options against illness caused by this microorganism.

In a study conducted by Tai et al. on *Salmonella* samples isolated from raw meat in Vietnam, the highest antibiotic resistance observed in *S*. Typhimurium was against tetracycline (86.4%), followed by streptomycin (81.8%) and nalidixic acid (77.3%) (39). In a study by Zeng et al., *S*. Typhimurium isolated from human clinical samples in China showed the highest resistance to ampicillin (81.6%), ciprofloxacin (81.6%), and tetracycline (82.4%) (40). These findings demonstrate the variation in *S*. Typhimurium resistance across different countries, highlighting the importance of resistance screening in each nation.

In the meta-analysis, Tadesse et al. indicated that certain strains of *Salmonella* found in farm animals in Ethiopia exhibit resistance to drugs commonly used for treating human salmonellosis, posing a potential risk of human exposure to drug-resistant *Salmonella* (41). Thung et al. conducted a study in Malaysia to investigate the presence of *Salmonella*, specifically *S*. Enteritidis and *S*. Typhimurium, in uncooked chicken meat (42). Their analysis of 120 chicken meat samples revealed that the prevalence of *Salmonella* and *S*. Typhimurium was 20.80 and 2.50%, respectively. Notably, all isolates exhibited resistance to erythromycin, penicillin, and vancomycin, with relatively lower resistance rates observed for nalidixic acid (9.09%) and streptomycin (9.09%). The study findings underscore the potential for chicken meat to serve as a reservoir of antibiotic-resistant *Salmonella*. Meanwhile, Talukder et al. conducted a meta-analysis focusing on the prevalence and antimicrobial resistance patterns of *Salmonella* strains isolated from human, animal, and environmental samples in South Asia (43). Their analysis revealed a lower prevalence of *salmonella* in humans (5.81%) compared to animals (22.66%) and the environment (27.81%). *Salmonella* strains displayed high resistance levels to nalidixic acid (74.25%) and tetracycline (37.64%), whereas lower resistance rates were observed for ceftriaxone (1.07%) and cefixime (1.24%). We categorized the origins of isolates into human, animal (such as poultry, livestock, and dogs), and food samples (including processed foods, meat, and eggs) for ampicillin, ciprofloxacin, nalidixic acid, tetracycline, gentamicin, and streptomycin. In all these antibiotics, the level of resistance in the samples isolated from animals was higher than in other sources. This emphasizes the importance of animal screening to check for the presence of drug-resistant isolates and the significance of controlling antibiotic consumption in animals to reduce antibiotic resistance and prevent the transmission of resistant strains to humans.

In general, third-generation cephalosporins and fluoroquinolones are considered the most effective and commonly used antibiotics for treating *S*. Typhimurium infections (44). The study findings revealed that among third-generation cephalosporins, cefixime, and ceftriaxone exhibited the lowest levels of resistance, making them favorable treatment options for infections caused by this bacterium. Additionally, within the fluoroquinolone class, norfloxacin demonstrated the least resistance compared to other antibiotics in this category, suggesting its potential as a treatment of choice for this bacterium in Iran. Furthermore, the resistance rate to imipenem in this bacterium was found to be 2%, indicating the potential usefulness of this antibiotic in managing MDR bacteria.

One limitation of the present study was the relatively high heterogeneity between studies. We employed subgroup analysis to identify sources of heterogeneity and mitigate its impact on the results.

## Conclusion

A high level of resistance to most of the studied antibiotics was observed. The highest antibiotic resistance was observed against piperacillin, tetracycline, doxycycline, nalidixic acid, and ampicillin, while cefixime, ceftriaxone, imipenem, and norfloxacin were the most effective treatment options for *S*. Typhimurium. Management and monitoring of antibiotic use, as well as screening to check the presence of drug-resistant isolates in animals, are recommended.

## Data availability statement

The original contributions presented in the study are included in the article/supplementary material, further inquiries can be directed to the corresponding author.

## Author contributions

NN: Conceptualization, Data curation, Investigation, Methodology, Software, Writing – original draft, Writing – review & editing. SR: Data curation, Methodology, Software, Writing – review & editing. FM: Data curation, Methodology, Software, Supervision, Writing – review & editing.
